# Biosafety and biosecurity challenges during the COVID-19 pandemic and beyond

**DOI:** 10.3389/fbioe.2023.1117316

**Published:** 2023-03-01

**Authors:** Saskia A. Rutjes, Iris M. Vennis, Edith Wagner, Vakhtang Maisaia, Lukas Peintner

**Affiliations:** ^1^ Laboratory for Zoonoses and Environmental Microbiology, Centre for Infectious Disease Control, National Institute for Public Health and the Environment (RIVM), Bilthoven, Netherlands; ^2^ Section of Experimental Virology, Institute of Medical Microbiology, Jena University-Hospital, Jena, Germany; ^3^ Faculty of Social Sciences, Caucasus International University, Tbilisi, Georgia; ^4^ Institute of Molecular Medicine and Cell Research, Albert Ludwigs University of Freiburg, Freiburg, Germany

**Keywords:** biosafety, biosecurity, online tools, emerging issues, low and lower middle-income countries

## Abstract

As the world continues to battle the SARS-CoV-2 pandemic, it is a stark reminder of the devastation biological threats can cause. In an unprecedented way the global community saw a massive surge in the demand for diagnostic capacities, which had a substantial impact on biosafety and biosecurity. Laboratories had to cope with a surge in laboratory testing capacity, while resources and training possibilities were limited. In addition, the pandemic highlighted the impact biological threats can have, thereby giving rise to new dialogue about biosecurity and new biological threats. This paper aims to highlight some of the most pressing issues regarding biosafety and biosecurity observed during the COVID-19 pandemic with special focus on low and lower middle-income countries. The authors provide lessons learned, tools and recommendations to improve future biosafety and biosecurity and increase preparedness for the next global health crisis.

## Introduction

The COVID-19 pandemic changed the world as we knew it. Not only our everyday life was profoundly shaken, also the way we perform and disseminate science faced massive overhauls. As demonstrated by the pandemic, it is essential that public health laboratories have the capacity to work safely and securely on emerging pathogens that can have high consequences. This is especially important for low and lower middle-income countries, classified by the World Bank as countries with a gross national income (GNI) *per capita* of $4,255 or less (World Bank). Due to the rapid spread of the disease around the globe and the excessive amount of potential infected patients, diagnostic laboratories faced a surge in specimen inflow. However, in the first months of the pandemic, certain characteristics of SARS-CoV-2 remained unknown and it lasted till May 2020 for the first laboratory biosafety guidance for SARS-CoV-2 to be published (WHO, 2019). New insights and developments during the pandemic led to changes in handling procedures (Kaufer et al., 2020; Naeem et al., 2022). This together with a massive growth in testing demand resulted in a series of biosafety and biosecurity issues.

Especially in the summer months of 2020 many laboratories and new established diagnostic facilities had to expand their capacities swiftly, often facing shortages in personal protective equipment and basic laboratory furniture.

Now in hindsight it is possible to identify three major topics, where biosafety and biosecurity policies may need to be adapted and improved to serve the laboratory manager and operative in a future pandemic. Those three topics are biosafety under resource limited conditions, training and communication of COVID-19 biosafety aspects, and biosecurity challenges under pandemic circumstances. The concept of biosafety is defined as the aggregate of measures, focusing on the prevention of an unintentional release of hazardous biological agents (World Health Organisation, 2020), and biosecurity as all measures focusing on the block of an intentional release of biological agents (National Research Council (US), 2009; Vennis et al., 2021; World Health Organization, 2006).

In this paper the authors describe their insights of issues and pitfalls in biosafety and biosecurity policies in practice observed in multiple countries and laboratories during the fight against the pandemic. It aims to foster a discussion on gaps and improvements in biosafety, biosecurity, and trainings by highlighting lessons learned and potential solutions.

## Biosafety under resource limited conditions

The emergence of a new pathogen or a zoonotic microbe that mutated and changed its host range needs a new classification of its risk level by established experts. Such scientific studies are performed in high or maximum containment laboratories that are usually operated by governmental institutions. However, since the construction and maintenance of such laboratories is very expensive, many low and lower middle-income countries are dependent on the information provided by resource rich countries. As was observed during the COVID-19 pandemic, such information on safe handling of the virus came quickly from various laboratories. In the course of the pandemic, scientific institutions constantly gained new insights and shared them in the form of peer-reviewed publications, but also as preprints under review to timely enclose the information to the scientific community. Many publishers of scientific literature understood their role in educating people and made relevant publications about the virus free of access [Callaway, 2020; Wellcome]. Nevertheless, official global bodies such as the WHO took up to 6 months after the start of the pandemic to establish a universal list of recommendations and best practices for the safe handling of viral diagnostics (Timeline, 2019; Maxmen, 2021). Because of this delay in access to official recommendations, laboratories had to make their own biosafety protocols with limited scientific knowledge about the properties of the virus.

A safe handling of microorganisms in the laboratory is based on its risk categorisation and a risk assessment. Accordingly, operators of laboratories can select from listed techniques and SOPs suitable for them, appropriate selection of Personal Protective Equipment (PPE, primary barrier), and whether the use of a Biosafety Cabinet (secondary barrier) is advised. In resource-limited environments, not every laboratory is fully equipped with the appropriate equipment and it is a management decision for which activities to allocate the limited machine-pool. Several low and lower-middle income countries reported to be struggling with the right safety equipment such as sufficient appropriate biosafety cabinets (Faust et al., 2020). In addition to a lack in official technical information and limited biosafety resources, the halting supply of COVID-19 vaccinations to low and lower middle-income countries made it further impossible for many laboratory operators to protect their staff.

A potential solution to that issues represents the ‘Sustainable Laboratories Initiative Prior Assessment Tool’ an online tool supporting laboratory managers in allocating funding and laboratory equipment that is provided by the Chatham House think tank (chathamhouse, 2019). This tool is meant to help structure a conversation between funding partners and recipient countries on how to most effectively establish or repurpose laboratories in low-resource environments. The medium provides a structure for a conversation between the funding partner and recipient country early in the process. It is based on a local risk assessment, whereby laboratories are appropriately and optimally tailored to the local risks and to the resources available, both in the short and longer term, without compromising biosafety and biosecurity. It seeks to increase local ownership and help partners ensure they have given due attention to all the relevant aspects, including risks and benefits, that need to be considered at an early stage. It should provide clarity on what is needed and improve the sustainability of any laboratory project that might result from the discussions. The tool contains questions regarding national strategic engagement, general framing of the laboratory and four essential functional aspects that should be considered prior to embarking on establishing or repurposing a laboratory: finance, human resources, operations, and infrastructure and utilities (chathamhouse, 2019).

An alternative *ad hoc* solution for countries struggling with a massive outbreak of a disease include the deployment of a mobile laboratory operated by several countries or state unions like the EU, WHO and others (Wölfel et al., 2015; EU CBRN CoE). Such mobile laboratories are designed to operate in resource-limited areas and are rapidly deployable. They contain equipment to perform basic diagnostic analysis on given pathogens and are intended to give a short time relief to governmental diagnostic laboratories until a stable operative infrastructure is built. However, mobile laboratories are very expensive to set up and are further dependent on highly qualified technical personnel. Policymakers from low and lower middle-income countries should know that many countries are operating such laboratories and are happy to support health systems in need. Nevertheless, the pandemic may serve as a wakeup call for many policymakers that the next global health crisis may be just around the corner and it needs funding and dedication from the governmental bodies to install the primary and secondary barriers to be physically prepared for the next outbreak.

## Training and communication of COVID-19 biosafety aspects

Next to the physical preparation of a country to raise its resilience against the next pandemic it is paramount to invest into highly qualified and reliable staff operating in the laboratories and performing the diagnostic testing.

Staff working in a certified ISO 15189 or 17025 laboratory regularly needs to attend advanced training courses to keep their certification (OECD, 1998; Zimmermann et al., 2019). Copious training courses are offered among others by several governmental institutions or non-profit organisations. However, the SARS-CoV-2 pandemic with its global traveling restrictions and the sudden need for more personnel brought such training to a standstill.

Over the timespan of a few months into the pandemic, many organisations started to offer online training courses. However, these solutions faced several challenges. Beside many technical hurdles that contained mostly limited access to computer hardware or insufficient internet connections, cultural challenges also had to be overcome. Offering such courses often faced difficulties to reach the correct audience. There is no point in teaching a laboratory manager the correct procedures in how to run a qPCR, when the technical staff never hears of this information. Hence, it was good to build on pre-existing networks and train the trainer initiatives to ensure the proper use of such online training courses. Several online initiatives by various national and international institutions were launched over the last 3 years. For instance, the German Biosecurity Programme funded by the German Foreign Office launched the “COVID-19 Digital Initiative”. This consists of two main components 1) a COVID-19 Information Hub, and 2) a series of COVID-19 related digital self-study modules. While the first provided a demand-driven selection of scientific publications and regular newsletters informing about advances in fighting the pandemic, the latter focused on virtual and practical laboratory training. In total, seven modules available in three languages (English, French and Russian) taught the basics on how to safely handle swabs samples, isolate viral RNA, and conduct WHO approved PCR screening (Peintner, 2023). While this course was created as a self-study initiative, other initiatives hired a designated teacher that informed their participants in their native language about biosafety measures regarding the handling of SARS-CoV-2 in the laboratory (Zimmermann et al., 2019).

Another example is the “Biosafety/Biosecurity Hybrid Train the Trainers Program in Georgia” organized by the Netherlands National Institute for Public Health and the Environment (RIVM), co-funded by the Dutch Ministry of Foreign Affairs and CBRN Centres of Excellence Project 53. This program, available in English and Georgian, taught Basic Laboratory Biosafety, Biorisk Assessment, Dual-Use, and how to train new trainers in a hybrid manner, starting with interactive online sessions, followed by in person training once the travel restrictions were released.

A completely different approach in supporting policymakers and lab operators are online decision-making tools. For example, the Netherlands Biosecurity Office has developed a toolkit that can help to increase biosecurity awareness (bureaubiosecurity). Besides an informative film, and gadgets to raise biosecurity awareness (postcards and the 10 golden security rules), the biosecurity toolkit also includes the ‘Biosecurity Self-scan Toolkit’ and the “Vulnerability Scan”. These are online tools to analyse biosecurity vulnerabilities in an organisation dealing with high consequence pathogens. Furthermore, as precise instructions for researchers on how to perform a dual-use risk assessment was largely lacking, the Biosecurity Office developed the “Dual-Use Quickscan”. This tool aims to identify potential dual-use aspects in research and contributes to stimulating dual-use awareness. Increased international attention to examine pathogens with pandemic potential has been enhanced by the COVID-19 pandemic, hence monitoring of dual-use potential needs to be encouraged (Vennis et al., 2021).

Moreover, Biosecurity Central is a publicly available web-based library that helps users find relevant and reliable sources of information for key areas of biosecurity. The site aims to widely disseminate and share knowledge to help advance biosafety and biosecurity. The library is a searchable and filterable database designed to enable ready access to biosafety and biosecurity resources from around the globe, published by governmental, international, and non-governmental organisations (Biosecurity Central). Table 1 provides multiple examples of tools that support biosafety and biosecurity.

**TABLE 1:** Some examples of tools to support education and outreach on biosafety and biosecurity topics. The tools listed are created and maintained by either governmental or non-governmental organizations and have the common goal of assisting life science researchers and laboratory managers in creating a safe work environment.

Name of tool	Content	Access
Biosecurity Central	-Laboratory biosafety -Legal mechanisms and authorities -Risk assessment -Laboratory biosecurity -High-consequence pathogens -Laboratory research -Dual use -Animal health, Zoonotic diseases -Law enforcement -Medical diagnostics -Export, Sample transportation -Environmental safety	https://biosecuritycentral.org
Surge Capacity Assessment Tool	E-learning and assessment questionnaire and calculator	https://lms.sckcen.elonisas.dev/moodle/login
Dual-Use Quickscan	Freely available webtool to assess dual-use potential of life science research	https://dualusequickscan.com
German Online Platform for Biosecurity and Biosafety (GO4BSB): ‘COVID-19 Digital Initiative’	-Newsletter -Wiki -Collection of publications -Self-study modules	www.go4bsb.de
Biosecurity pillars of good practice	Basics of working with high risk biological material	https://www.bureaubiosecurity.nl/en/node/531
Biosecurity Vulnerability Scan	Helps to detect weak spots in laboratory security. An extensive scan with questions, scenarios and best practices built around the eight pillars of biosecurity	https://www.biosecurityvulnerabilityscan.nl
Biosecurity Self-scan Toolkit	A relatively fast scan with a limited number of closed questions that can easily form an indication of strong and weak biosecurity aspects within your organisation	https://biosecurityselfscan.nl
Ten golden rules of biosecurity and biosafety	Explains the cornerstones of biosecurity	https://www.bureaubiosecurity.nl/en/information/10-golden-rules-of-security
Biosecurity Checklist	Laboratory Biosecurity Assessment and Monitoring Checklist for biosecurity monitoring and auditing of laboratories	https://www.liebertpub.com/doi/10.1177/1535676019838077 Brizee et al., 2019
International Gene Synthesis Consortium	A common protocol to screen DNA sequences	https://genesynthesisconsortium.org/
Basic cybersecurity measures	Several cases exploring potential cybersecurity issues	https://english.ncsc.nl/

The COVID-19 pandemic has brought about preventive measures that have had a considerable impact on various dimensions of biosafety and biosecurity teaching and learning. While digital teaching and learning approaches cannot substitute in-person training, they have shown to be useful tools to complement other training formats, and can provide guidance during outbreak of newly emerging pathogens, such as SARS-CoV-2.

## Biosecurity challenges under pandemic circumstances (in regard of physical and cybersecurity aspects)

The rise of the SARS-CoV-2 pandemic has sparked public interest in the biological sciences. In contrast to before the pandemic, non-professionals became familiar with concepts of incidence rates, incubation periods, herd immunity, vaccinations and PCR testing. In addition, the pandemic initiated new discussions about weaponization of biological entities and biosecurity gained new momentum (CTPN, 2021). Although the use of microorganisms and toxins as weapons is strictly prohibited by the Biological and Toxin Weapons Convention (BTWC) and United Nations Security Council Resolution 1540 (UNSCR1540), some think tanks see a potential rise in the interest of individual states of starting and pursuing new biological weapons initiatives. The Washington D.C. based council of strategic risks envisions three potential scenarios developing from the COVID-19 crisis (Bajema et al, 2022). In Scenario one they claim that the damage caused by COVID-19 leads to the rise of biological weapons as a significant component of deterrence for many nations, with these trends intersecting and feeding into greater security tensions. Scenario two envisions the exact opposite and predicts that fear of future biological threats bolsters international cooperation—states are driven to avoid another catastrophic biological event, working together to better utilise technologies and enhance diplomatic mechanisms. In Scenario 3 the think tank combines these two aforementioned scenarios and envisions a lack of momentum after the current pandemic translates into weak progress in strengthening healthcare systems, waning interest in developing global early warning systems, and a continued rise of biological threats. They claim that these scenarios may help policymakers by illustrating the plausible ways biological weapons could shape global affairs—and in turn, provide the foresight needed to make decisions and investments that avoid the worst of these realities.

Other institutions like the European Center of Disease Control (ECDC) sees the biggest danger in new forms of terrorism. Now the public is aware of the threats posed in a (zoonotic) outbreak (Episode 23 - Paul Riley—Bioterrorism and biosecurity). Terrorists could instrumentalize these fears and cause mass panic among citizens. Even though terrorists are probably not able to successfully build and deploy biological warheads, the simple spraying of bacteria or viruses in a densely populated area or the poisoning of drinking water would be enough to terrify the public. There are also stories of panic caused by excessive faked coughing in a public gathering to disturb a political discussion (Arora et al., 2020). Bioterrorism should be seen as one of the new asymmetric challenges of the contemporary international security environment with the aim to impose concrete political, ideological and quasi-religious opinions mainly by non-state aggressive actors (Maisaia and Alika, 2020).

Although most terrorists are unlikely to be able to build a biological weapon, bioscientists do have the necessary skills. One of the greatest threats to the successful misuse of microorganisms is therefore rogue scientists, who pose a potential insider threat (Perkins and Fabregas, 1773). The fight against insider threat is largely based on personnel reliability. Insiders with fraudulent intent can look up information and have access to high consequence pathogens easily as they have been granted access to databases and pathogen inventories. Hence it is paramount to perform an in-depth security check of all existing and new employees in an institution that is handling sensitive information. One initiative to screen the activities of scientists rests in the surveillance on the orders of primers and gene sequences by the ‘International Gene Synthesis Consortium (IGSC)’ (IGSC, 2017). With regard to research with the virus and the production of (parts of) SARS-CoV-2, there are guidelines for ordering synthesised viral sequences (*e.g.*, primers for PCR). The IGSC is an industry-led group of gene synthesis companies and organisations and has established a “Harmonised Screening Protocol” to prevent abuse of synthetically produced sequences. It is their aim to protect the positive aspects of gene synthesis technology while minimising the risk of misuse.

Most life scientists probably do not have malicious intents, but it is important that they have sufficient awareness about biosafety and biosecurity to work safe and securely. For example, it is crucial for employees to be aware to never leave data unprotected and unattended. Still one of the most common ways to get behind the firewall of databases are phishing programmes on USB sticks or E-mail attachments. The best digital countermeasures can be easily bypassed by the thoughtlessness of the employees (Ferreira and Cruz-Correia, 2021; Mueller, 2021). As these examples demonstrate, security in the biological sciences is expanding to the cyberspace. Hence, in the last decade the term cyberbiosecurity was termed (Adler et al., 2021). Richardson et al. describe cyberbiosecurity as *“addresses the potential for or actual malicious destruction, misuse, or exploitation of valuable information, processes, and material at the interface of the life sciences and digital worlds*" (Richardson et al., 2019). Key issues of concern include, among others, the privacy of patient data, the security of public health databases, the integrity of diagnostic test data, the integrity of public biological databases, the security implications of automated laboratory systems and the security of proprietary biological engineering advances.

But, as already briefly mentioned above, cyberbiosecurity does not only concern the public health sector but amongst others also the field of synthetic biology. Technologies in synthetic biology were rapidly advancing over the last decade and genetic sequences were openly published. With the new techniques and public genetic information whole stretches of sequences can be produced artificially. Even a bigger threat is the possibility of cyber-criminals remotely injecting malicious DNA sequences, resulting in life scientist unknowingly developing biological threats (Puzis et al., 2020). Another cyberbiosecurity example is the possibility to hack a negative pressure system with the aim to breach containment of dangerous pathogens. Researchers in the US sought to probe whether negative pressure systems could be hacked and succeeded (Poste and Gillum, 2023). This highlights the need for robust cybersecurity measures to protect vital healthcare infrastructure during a public health emergency.

During the COVID-19 pandemic, there were multiple reported cases of cyberattacks targeting healthcare organizations, including hospitals and research institutions. These attacks aimed to disrupt operations and steal sensitive information, such as patient data and research findings. Further, as a result of the pandemic, many organizations shifted to remote work, which increased the risk of cyberattacks such as phishing, malware and other forms of cybercrime. In 2020, several hospitals in India reported cyberattacks that disrupted their operations, including the theft of patient data (AFP, 2022; Wasserman and Wasserman, 2022) and also Brazil reported an increase in cybercrime, including phishing scams and ransomware attacks targeting individuals and organizations, including healthcare providers (Macedo and Singleton). In Africa there have been numerous reported cases of cybercrime targeting individuals and organizations in different African countries during the pandemic, including phishing scams, malware, and ransomware attacks (Chigada and Madzinga, 2021). These attacks took advantage of the increased reliance on digital systems during the pandemic, highlighting the need for improved cybersecurity measures, especially in healthcare organizations in low and lower-middle income countries.

The impact of the pandemic on biosecurity is discussed on many levels. WHO, for instance, aims to publish a new laboratory biosecurity guidance for biorisk management in the beginning of 2023 (Kojima, 2022), as the latest edition dates from 2006 (World Health Organization, 2006). The WHO saw that after the pandemic there is a need to develop a global minimum requirement for safeguarding global health security. WHO is calling for a consensus definition of global minimum requirements focused on biological risk management of laboratory activities. They call for consensus-based standards developed for global best practices, not to replace them. These claims follow three rationales: First, WHO identifies growing concerns for biosafety and biosecurity. WHO recommends their “WHO BioHub system biosafety and biosecurity: Criteria and operational modalities” (World Health Organization, 2022). Second, WHO calls for a review of existing legislation. They ask if the current national legislations are enough to prevent various scenarios? Finally, WHO wants to increase the focus on the Biological and Toxin Weapons Convention (BTWC). They call for a verification mechanism based on ISO35001 with a neutral third party assessment for safe and secure operations (ISO 35001:2019, 2019).

In addition, international initiatives such as the Global Health Security Agenda (GHSA), Global Biosecurity Dialog (GBD), and the Global Partnership Against the Spread of Weapons and Materials of Mass Destruction (GPWMD) play a major role in building biosecurity capacity and employing international legally binding biosecurity instruments (Vennis et al., 2022). Such legal instruments, as the BTWC (disarmament) and UNSCR1540 (UN Security Council, 1540) are international legally binding non-proliferation instruments to reduce dangers of deliberate disease outbreaks in humans, animals and plants. The BTWC also contributes to global disease surveillance as it requests international exchange of equipment, materials, and information to combat outbreaks of infectious diseases. UNSCR1540 emphasises safe and secure handling, use, transport, and storage of pathogenic material, thereby contributing to biosafety and biosecurity. Furthermore, the COVID-19 pandemic has increased attention toward the WHO’s International Health Regulations 2005 (IHR) (World Health Organisation, 2005). IHR focusses on infectious disease outbreaks with a natural origin and covers some aspects of accidental and deliberate releases. However, independent of the origin of a disease outbreak, an effective public health response is necessary to control it.

(Vennis et al., 2022) identified overlapping and complementary issues in IHR, UNSCR1540 and BTWC with the aim to improve understanding of policymakers, civil servants, biosecurity experts, and practitioners regarding these instruments. This accommodates the enhancement of full employment of national resources to comply with international requirements, ultimately leading to an improved capacity to prevent, detect and respond to infectious disease outbreaks, independent of their origin.

## Lessons learned and suggestions for improvements

As with the corona pandemic, previous outbreaks also highlighted weaknesses in laboratory preparedness. One of the examples of laboratory shortcomings during the SARS outbreak (2002–2004) are the reports on laboratory acquired infections in China and Singapore (Lim et al., 2004; WHO, 2004). The SARS outbreak demonstrated there are unforeseeable threats, whether natural emerging diseases or biosecurity threats. After SARS, the International Health Regulations (IHR) were revised with the aim to prevent and control public health threats while avoiding unnecessary interference with international travel and trade. The revised regulations included *“all events potentially constituting a public health emergency of international concern (PHEIC)”* (CDC, 2019). Monitoring and evaluation of IHR was mainly through the States’ Self-Assessment Annual Report (SPAR). The Ebola outbreak (2014–2016) clearly demonstrated that this self-reporting mechanism did not provide an accurate representation of IHR implementation. The countries concerned with Ebola had reported rather high levels of implementation, which appeared to be an overestimation once facing the outbreak. After the Ebola outbreak the JEEs (joint external evaluations) were established to move from exclusive self-evaluation to approaches that combine self-evaluation, peer review and voluntary external evaluations involving a combination of domestic and independent experts (WHO, 2005). In October 2019, the Global Health Security Index analysis found no country to be fully prepared for epidemics or pandemics (Vennis et al., 2022). The COVID-19 pandemic demonstrated that the world collectively indeed did not have sufficient capacity to prevent and control major infectious disease outbreaks, as also shown in the 2021 Global Health Security Index report. The report found “Although many countries were able to quickly develop capacities to address COVID-19, all countries remain dangerously unprepared for meeting future epidemic and pandemic threats.” Towards the end of the pandemic statements were made that the IHR “are a conservative instrument that constrain rather than facilitate rapid action” (Sirleaf and Clark, 2021). WHO established a Review Committee on the Functioning of the International Health Regulations (2005) during the COVID-19 Response. The committee summarized that the IHR can certainly facilitate adequately, but many countries only applied the IHR in part and that WHO did not make fully use the established powers they have (WHO, 2021). The COVID-19 pandemic and previous outbreaks demonstrate that many international efforts were made to adhere to an international standard of preparedness. However, both the Ebola outbreak and COVID-19 pandemic clearly show that implementation of IHR in practise is still a weakness.

Furthermore, many countries, both low and lower middle-income countries and resource rich countries, faced difficulties to keep an overview on the maturing body of SARS CoV-2 knowledge, including biosafety and biosecurity measures. Still, it was apparent that many low and lower middle-income countries struggled to have equal access to diagnostic tools, safety equipment, training, and vaccine supply. Hence, it needs to be the focus of the global community to prepare for these issues in non-pandemic times. There is a need for a strategy on how to train more laboratory specialists, so that they are readily available in the next pandemic and to install a viable global stockpiling system of diagnostic materials and laboratory equipment to supply all countries equivalent.

So far, this paper elaborated on biosafety and biosecurity standards in public health, since that was the field that got challenged the most during the COVID-19 pandemic. However, SARS CoV-2 is a zoonotic disease and the hunt for the species that finally transduced the virus from animals to humans is still ongoing (Lytras et al., 2021). Hence, biosafety and biosecurity in the area of animal health play a critical role in preventing and controlling veterinary disease outbreaks that can pose significant risks to public health and the economy. Effective biosafety and biosecurity measures are crucial in preventing and controlling the spread of diseases in animals and reducing the risk of transmission to humans. The WHO propagates this in an one-health approach (WHO), however, in low and lower middle-income countries farmers and meat production companies often face the issue of a lack of resources such as funding, trained personnel, and infrastructures for animal health (Future of Animal Science Research, 2015). These existing infrastructures may not meet the necessary biosafety and biosecurity standards (Siengsanan-Lamont et al., 2019). This includes facilities for housing and caring for animals, as well as laboratories for disease diagnostics. These deficits in the hardware can be potentiated with a lack of awareness and education among relevant personnel and farmers about the importance of biosafety and biosecurity in animal health, and the measures that need to be taken to prevent and control disease outbreaks.

To address the above mentioned challenges, it is important to invest in building the necessary resources and infrastructure in a one-health setting, as well as in increasing awareness, education, and training about the importance of biosafety and biosecurity measures (Butucel et al., 2022). Additionally, international cooperation and collaboration are essential in sharing knowledge, best practices and resources to improve the implementation of these measures, particularly in low and lower middle-income countries. Furthermore, the authors argue that international regulations are important, but biorisk management could benefit from more emphasis on practical implementation of biosafety and biosecurity policies.

## Conclusion

The ongoing COVID-19 pandemic highlights the need for laboratories that have the capacity to work safely and securely with emerging pathogens. International instruments from different disciplines address these health and security challenges, setting requirements for states to effectively prevent, detect, and respond to infectious disease outbreaks, either with deliberate or non-deliberate origin (Vennis et al., 2022).

In this policy and practice review the authors intended to highlight some of the initiatives that aim to tackle biosafety-, biosecurity- and training concerns provoked by the pandemic. However, the pandemic is only slowly coming to an end and it will take many more years to fully understand the impact of this event on how we will perform safe and secure science and diagnostics in the future.
